# Tryptophan metabolism in liver transplantation immune tolerance

**DOI:** 10.3389/fimmu.2026.1873690

**Published:** 2026-07-16

**Authors:** Wei Li, Ao Ren

**Affiliations:** Department of Hepatobiliary Surgery, The First Affiliated Hospital of Chongqing Medical University, Chongqing, China

**Keywords:** IDO, immune tolerance, liver transplantation, TDO2, tryptophan metabolism

## Abstract

Liver transplantation is a life-saving treatment for end-stage liver disease, but long-term outcomes are limited by complications of lifelong immunosuppression. Inducing immune tolerance has become a major research priority. Tryptophan (Trp) metabolism, particularly the kynurenine pathway, is a key endogenous regulator of peripheral tolerance. This review moves beyond a simple description of metabolic routes and provides a critical, integrated analysis. We systematically compare the non−redundant immunosuppressive roles of indoleamine 2,3−dioxygenase (IDO) and tryptophan 2,3−dioxygenase (TDO2) in liver transplant immunity, dissect the Trp/kynurenine/AhR pathways that reprograms immune cell function, and incorporate gut−microbiota−dependent indole metabolism as an upstream gut−liver axis node. Clinical evidence is stratified by level, and major translational barriers, including safety risks, delivery challenges, and lack of validated biomarkers are discussed. Current data suggest that combined metabolite panels may outperform single markers, but therapeutic targeting of Trp metabolism remains a preclinical research direction rather than an established therapy.

## 1 Introduction

Liver transplantation is a life-saving intervention for patients with end-stage liver disease, severe hepatitis, and liver malignancies (1). Advances in surgical techniques, organ preservation, and immunosuppressive regimens have markedly improved short-term survival. Nevertheless, long-term prognosis remains constrained by persistent challenges, including acute and chronic allograft rejection, nephrotoxicity from prolonged immunosuppression, infectious complications, and tumor recurrence (2, 3).

The liver is an immunologically privileged organ in which immune responses are biased toward tolerance rather than activation (4–6). Within this unique microenvironment, tryptophan (Trp) metabolism has emerged as a key endogenous regulator of peripheral tolerance (7–9). Accumulating evidence indicates that indoleamine 2,3-dioxygenase (IDO), tryptophan 2,3-dioxygenase (TDO2), and their metabolites (e.g., kynurenine (Kyn) and indole derivatives) participate in the induction and maintenance of immune tolerance after liver transplantation by suppressing effector immune cells, promoting regulatory cell generation, and remodeling the intrahepatic immune microenvironment (10–12).

Distinct from previous reviews that mainly focus on the function of IDO and TDO in tumors and autoimmune diseases, or simply summarize tryptophan metabolic pathways, this review systematically elaborates the respective and unique regulatory roles of IDO and TDO2 in liver transplantation immunity (13, 14). Furthermore, it dissects the molecular mechanism by which the sequential signaling cascade of tryptophan depletion, kynurenine accumulation, and aryl hydrocarbon receptor (AhR) activation reprograms immune cell function. In addition, this paper analyzes the role of microbiota-mediated indole metabolism, as an upstream regulatory node of the gut-liver axis, in immune tolerance following liver transplantation. Finally, relevant issues regarding clinical translation are discussed, so as to lay a theoretical foundation for subsequent mechanistic research and clinical investigation.

## 2 The role of tryptophan metabolism in LT immune tolerance

### 2.1 IDO and TDO2

Tryptophan metabolism is initiated by three rate-limiting enzymes: IDO1, IDO2, and TDO2 (15, 16). TDO2 exhibits a tissue-specific expression pattern, predominantly localized in the liver, whereas IDO isoforms are widely distributed across multiple organs, including the brain and gastrointestinal tract (17). IDO expression is inducible by various pro-inflammatory mediators, such as interferon-gamma (IFN-γ) and tumor necrosis factor-alpha (TNF-α), with the serum kynurenine/tryptophan (Kyn/Trp) ratio serving as a reliable biomarker for assessing IDO enzymatic activity (18, 19).

Preclinical studies have shown that IDO upregulation correlates with spontaneous graft tolerance. Miki et al. (20) demonstrated in a mouse liver allograft model (C57BL/10j → C3H) that IDO mRNA peaked on day 7 and remained elevated until day 30. Administration of the IDO inhibitor 1−methyl−tryptophan (1−MT) on day 0-–but not on day 7-–abrogated tolerance, leading to acute rejection within 13 days. This functionally established IDO−mediated tryptophan catabolism as a mechanism of liver immune privilege. Similarly, Lin et al. (21) showed that IDO is induced in antigen-presenting cells of hepatectomized livers by which subsequently transplanted cells may be protected from rejection by inhibiting indirect or direct recognition of donor antigen and further T-cell activation.

However, IDO overexpression is not universally protective. Laurence et al. (22) used an rAAV vector to overexpress IDO specifically in hepatocytes in a rat (PVG−to−LEW) liver transplant model. Although serum tryptophan fell to half of baseline, allograft survival was not prolonged (median survival 12–13 days, comparable to controls). This apparent contradiction highlights key considerations: cell type (hepatocytes vs. antigen−presenting cells), the spatiotemporal pattern of tryptophan depletion, and the timing of intervention may determine efficacy. Standalone IDO overexpression in parenchymal cells may be insufficient to counter the complex alloimmune response, whereas IDO activity in professional antigen−presenting cells such as dendritic cells (DCs) and Kupffer cells appears to be more effective (23–25).

In contrast, TDO2 has only recently been investigated in the context of liver transplantation. Li et al. (26) generated TDO2−knockout rats using CRISPR−Cas9. In a Lewis−to−Brown Norway (BN) rejection model, TDO2 deficiency exacerbated acute rejection, shortened recipient survival (median survival reduced from >30 days to approximately 14 days), increased pro−inflammatory cytokines (IFN−γ, TNF−α), and decreased tolerogenic cytokines (IL−4, IL−10). Multiplex immunohistochemistry revealed that TDO2 knockout led to a reprogrammed immune cell distribution: increased CD8+ T cells and M1 macrophages in portal areas, and decreased regulatory T cells (Tregs) and M2 macrophages in intermediate lobular regions. These findings suggest that TDO2 and IDO play complementary, non−redundant immunosuppressive roles in liver transplantation: IDO is more relevant in antigen−presenting cells and can be induced by inflammation, whereas TDO2 constitutively metabolizes Trp in hepatocytes and may contribute to baseline immune regulation.

### 2.2 The Kyn−AhR axis: connecting catabolism to immune reprogramming

Kynurenine is not merely a waste product; it is an endogenous AhR ligand. The Kyn−AhR axis is the central bridge connecting tryptophan catabolism to immune cell reprogramming (27, 28). *In vitro* and *in vivo*, kynurenine activates AhR, promoting naive CD4+ T cell differentiation into Foxp3+ Tregs while suppressing Th17 polarization (29, 30). Using multiplex immunohistochemistry, Li et al. (26) showed that TDO2 knockout led to reduced AhR target gene *CYP1B1* expression, accumulation of CD8+ T cells and M1 macrophages in portal areas, and decreased Tregs and M2 macrophages in intermediate lobular regions. Exogenous kynurenine reversed the effects of TDO2 knockdown in coculture experiments, confirming that the Kyn−AhR axis is essential for the tolerogenic function of TDO2.

Further supporting this axis, IFN−γ−treated IDO−competent DCs have been shown to attenuate acute rejection in rat liver transplantation by enhancing IDO expression, increasing Kyn production, and promoting lymphocyte apoptosis (31). Conversely, pharmacological inhibition of IDO with 1−MT abrogates spontaneous liver allograft tolerance in mice (20), demonstrating that IDO−mediated Trp catabolism is critical for maintaining graft acceptance. Thus, TDO2/IDO−derived kynurenine, via AhR, reprograms T−cell and macrophage lineage decisions, creating the tolerogenic characteristic within the graft.

The gut microbiota adds another layer of regulation. Gut dysbiosis, which is common in many chronic liver diseases and also occurs early after liver transplantation, directly impairs tryptophan metabolic efficiency (32, 33). Gut bacteria such as Clostridium, Bacteroides, Bifidobacterium, and Lactobacillus metabolize dietary Trp into various indole derivatives, including indole−3−aldehyde, indole−3−acetic acid, indole−3−propionic acid, and indole−3−carboxylic acid (34–36). Many of these indole derivatives are also AhR ligands and can modulate intrahepatic immune responses after being absorbed and transported to the liver via the portal vein (37, 38). Although most current evidence comes from animal models or *in vitro* studies, this gut−liver axis represents a novel regulatory node for immune modulation in liver transplantation. However, direct causal evidence linking specific microbial indole metabolites to graft tolerance in humans is still lacking.

## 3 Clinical value of tryptophan metabolism in liver transplantation immune tolerance

Currently, clinical evidence comes mainly from small observational studies. Ingelsten et al. (39) studied eight highly sensitized patients undergoing combined auxiliary liver−kidney transplantation. At 4 hours post−reperfusion, IDO mRNA in the liver allograft was upregulated 12−fold, reaching 93−fold at 1 week, preceded by increases in TNF−α, IL−1β, and IFN−γ. Interestingly, the kidney allograft from the same donor also showed 86−fold IDO upregulation at 1 week, suggesting that the liver graft may release soluble factors exerting a “tolerogenic radiation” effect on distant organs. This phenomenon may explain why combined liver−kidney transplantation sometimes leads to better kidney graft acceptance in highly sensitized recipients.

As a biomarker, the serum kynurenine/tryptophan ratio, an indirect measure of IDO activity, has been associated with acute rejection in kidney transplantation (40, 41). In liver transplantation, limited data suggest it may help assess post−transplant immune status, but no standardized cut−off values exist, and prospective cohort validation is lacking (42). Single−metabolite measurements may be insufficient; combined panels including kynurenine, tryptophan, indole derivatives, and inflammatory markers may offer higher predictive value, but this requires clinical validation.

Preclinical exploration of IDO−based therapy has yielded contrasting results. Sun et al. (31) generated IFN−γ−primed IDO−competent DCs and administered them in rat liver transplantation models, observing attenuated acute rejection and prolonged survival (median survival increased from 12 days to 27 days). In contrast, as mentioned above, rAAV−mediated IDO overexpression in hepatocytes failed to prevent rejection (22). These discrepancies emphasize that the choice of cell type (DCs vs. hepatocytes) and the mode of IDO delivery (cellular vs. gene therapy) critically influence outcomes.

TDO2 has not yet been directly targeted therapeutically, but its deficiency worsens rejection, suggesting that maintaining or enhancing TDO2 activity might be beneficial (43). Complementary research targeting downstream metabolites of the kynurenine pathway also supports translational potential. Xu et al. (44) demonstrated that 3,4−dimethoxycinnamonylanthranilic acid (3,4−DAA)-–a synthetic analog of anthranilic acid-–alleviated acute graft−versus−host disease (aGVHD) severity, prolonged recipient survival, and modulated T−cell polarization by suppressing Th1 cytokines (IFN−γ, IL−2, TNF−α) while promoting Th2 cytokines (IL−4, IL−10). Although this study was performed in a bone marrow transplantation model, it supports the concept that tryptophan metabolites themselves can be directly immunomodulatory.

Despite these compelling preclinical findings, several major barriers must be overcome before tryptophan−targeted strategies can enter clinical practice.

First, safety is a major concern. Systemic activation of IDO/TDO2 or administration of kynurenine analogs in transplant recipients could increase the risk of opportunistic infections (by suppressing protective immunity) (45, 46). Any strategy that broadly depletes Trp or elevates kynurenine must be carefully evaluated in the context of long−term immunosuppression. Additionally, certain tryptophan metabolites (e.g., 3−hydroxykynurenine, quinolinic acid) are neurotoxic and can cause neuronal degeneration if they accumulate systemically (47).

Second, targeted delivery remains a challenge. An ideal strategy would achieve local immunosuppression within the graft without systemic immune exhaustion. Liver−specific delivery systems for IDO activators, TDO2 modulators, or tryptophan metabolites are not yet available. Cell−based approaches (e.g., IDO−competent DCs) offer some selectivity but face scalability and reproducibility hurdles (31). The failure of rAAV−mediated hepatocyte−specific IDO overexpression suggests that simply increasing Trp catabolism in parenchymal cells is insufficient (22); the site and cellular context of IDO/TDO2 activity are critical.

Third, inter−individual variability is substantial. Differences in gut microbiota composition, genetic polymorphisms of IDO and TDO2, and baseline immune status may all affect the efficacy of Trp−targeted interventions (48, 49). For example, the IDO inhibitor 1−MT showed varying effects in different mouse strain combinations (20), suggesting that genetic background influences the contribution of IDO to tolerance. Validated stratification biomarkers are currently lacking, making personalized implementation difficult.

Therefore, targeting tryptophan metabolism should currently be viewed as a potential research direction, not an established therapy.

## 4 Conclusion

This review elaborates on the mechanisms and translational potential of tryptophan metabolism in liver transplant tolerance. The kynurenine pathway acts as the key connection between metabolic depletion and immune cell reprogramming, with IDO and TDO2 exerting distinct immunosuppressive effects; the Kyn-AhR axis further shapes a tolerogenic microenvironment, while gut microbiota-related indole metabolism, an upstream gut-liver axis component, still lacks solid clinical evidence. Relevant human research is in its infancy, as the kynurenine/tryptophan ratio and other biomarkers await standardization and validation, and combined marker panels are expected to perform better. Additionally, therapies targeting tryptophan metabolism face safety, delivery and cell-type specificity issues. Overall, though tryptophan metabolism is a promising field for liver transplant immunology, substantial challenges remain for its clinical translation.
